# Determinants of B-Cell Compartment Hyperactivation in European Adolescents Living With Perinatally Acquired HIV-1 After Over 10 Years of Suppressive Therapy

**DOI:** 10.3389/fimmu.2022.860418

**Published:** 2022-03-31

**Authors:** Alessandra Ruggiero, Giuseppe Rubens Pascucci, Nicola Cotugno, Sara Domínguez-Rodríguez, Stefano Rinaldi, Alfredo Tagarro, Pablo Rojo, Caroline Foster, Alasdair Bamford, Anita De Rossi, Eleni Nastouli, Nigel Klein, Elena Morrocchi, Benoit Fatou, Kinga K. Smolen, Al Ozonoff, Michela Di Pastena, Katherine Luzuriaga, Hanno Steen, Carlo Giaquinto, Philip Goulder, Paolo Rossi, Ofer Levy, Savita Pahwa, Paolo Palma, the EPIICAL Consortium, Mark Cotton

**Affiliations:** ^1^ Academic Department of Pediatrics (DPUO), Research Unit of Clinical Immunology and Vaccinology, Bambino Gesù Children’s Hospital, IRCCS, Rome, Italy; ^2^ Department of Neuroscience, Biomedicine and Movement Sciences, University of Verona, Verona, Italy; ^3^ Chair of Pediatrics Department of Systems Medicine, University of Rome ‘‘Tor Vergata’’, Rome, Italy; ^4^ Pediatric Research and Clinical Trials Unit (UPIC), Instituto de Investigación Sanitaria Hospital 12 de Octubre (IMAS12), Madrid, Spain; ^5^ Fundación para la Investigación Biomédica del Hospital 12 de Octubre, RITIP (Traslational Research Network in Pediatric Infectious Diseases), Madrid, Spain; ^6^ Department of Microbiology and Immunology, University of Miami Miller School of Medicine, Miami, FL, United States; ^7^ Department of Pediatrics, Infanta Sofía University Hospital. Infanta Sofia University Hospital and Henares University Hospital Foundation for Biomedical Research and Innovation (FIIB HUIS HHEN), San Sebastián de los Reyes, Madrid, Spain; ^8^ Universidad Europea, Madrid, Spain; ^9^ Department of Pediatric Infectious Diseases, Imperial College Healthcare NHS Trust, London, United Kingdom; ^10^ MRC Clinical Trials Unit at UCL, London, United Kingdom; ^11^ Great Ormond Street Hospital for Children NHS Trust, London, United Kingdom; ^12^ University College London Great Ormond Street Institute of Child Health, London, United Kingdom; ^13^ Department of Oncology, Surgery and Gastroenterology, University of Padova, Padova, Italy; ^14^ Istituto Oncologico Veneto (IOV)- IRCCS, Padova, Italy; ^15^ Infection, Immunity & Inflammation Department, UCL GOS Institute of Child Health, London, United Kingdom; ^16^ Precision Vaccines Program, Boston Children Hospital, Boston, MA, United States; ^17^ Harvard Medical School, Boston, MA, United States; ^18^ Department of Pathology, Boston Children’s Hospital, Boston, MA, United States; ^19^ UOSD Unit of Clinical Psychology – Dept. of Neuroscience and Neurorehabilitation, Bambino Gesù Children’s Hospital, IRCCS, Rome, Italy; ^20^ Program in Molecular Medicine, Umass Chan Medical School, Worcester, MA, United States; ^21^ Department of Mother and Child Health, University of Padova, Padova, Italy; ^22^ Department of Paediatrics, University of Oxford, Oxford, United Kingdom

**Keywords:** T-bet, CD11c, perinatal HIV/AIDS, B-cell hyperactivation, proteomic profiling immune activation, late ART, exhausted T-cells, caHIV-1 RNA

## Abstract

**Background:**

Despite a successful antiretroviral therapy (ART), adolescents living with perinatally acquired HIV (PHIV) experience signs of B-cell hyperactivation with expansion of ‘namely’ atypical B-cell phenotypes, including double negative (CD27-IgD-) and termed age associated (ABCs) B-cells (T-bet+CD11c+), which may result in reduced cell functionality, including loss of vaccine-induced immunological memory and higher risk of developing B-cells associated tumors. In this context, perinatally HIV infected children (PHIV) deserve particular attention, given their life-long exposure to chronic immune activation.

**Methods:**

We studied 40 PHIV who started treatment by the 2^nd^ year of life and maintained virological suppression for 13.5 years, with 5/40 patients experiencing transient elevation of the HIV-1 load in the plasma (Spike). We applied a multi-disciplinary approach including immunological B and T cell phenotype, plasma proteomics analysis, and serum level of anti-measles antibodies as functional correlates of vaccine-induced immunity.

**Results:**

Phenotypic signs of B cell hyperactivation were elevated in subjects starting ART later (%DN T-bet+CD11c+ p=0.03; %AM T-bet+CD11c+ p=0.02) and were associated with detectable cell-associated HIV-1 RNA (%AM T-bet+CD11c+ p=0.0003) and transient elevation of the plasma viral load (spike). Furthermore, B-cell hyperactivation appeared to be present in individuals with higher frequency of exhausted T-cells, in particular: %CD4 TIGIT+ were associated with %DN (p=0.008), %DN T-bet+CD11c+ (p=0.0002) and %AM T-bet+CD11c+ (p=0.002) and %CD4 PD-1 were associated with %DN (p=0.048), %DN T-bet+CD11c+ (p=0.039) and %AM T-bet+CD11c+ (p=0.006). The proteomic analysis revealed that subjects with expansion of these atypical B-cells and exhausted T-cells had enrichment of proteins involved in immune inflammation and complement activation pathways. Furthermore, we observed that higher levels of ABCs were associated a reduced capacity to maintain vaccine-induced antibody immunity against measles (%B-cells CD19+CD10- T-bet+, p=0.035).

**Conclusion:**

We identified that the levels of hyperactivated B cell subsets were strongly affected by time of ART start and associated with clinical, viral, cellular and plasma soluble markers. Furthermore, the expansion of ABCs also had a direct impact on the capacity to develop antibodies response following routine vaccination.

## Introduction

HIV-1 replication is associated with abnormalities in all major lymphocyte populations, including the B-cell compartment which results in hyperactivation and exhaustion (1–5). While early antiretroviral therapy (ART)-initiation partially averts this detrimental condition (6), late ART initiation during the chronic stage of HIV infection results in hyperactivation of the immune system with the expansion of exhausted B cell subsets, including activated memory (AM), double negative (DN)- and tissue-like memory B cells (TLM) (1, 4, 6, 7). In addition, recent studies have shown the presence of a particular subset of B-cells characterized by the surface expression of the transcription factor T-bet and the adhesion molecule CD11c and the so-called termed age associated B-cells (ABCs) (8, 9). This particular B-cell subset is physiologically induced by infecting agents, and it is found to be increased in settings of chronic stimulation triggered by self and non-self antigens (8–10). Overall, chronic B cell activation observed during HIV infection has been related to a reduction of functional resting memory B cells resulting in precocious waning of routine vaccine-induced antibody titers (11–13) and increased risk of age-associated pathologies (14, 15), including malignancies (16). Indeed, a B cell lymphoproliferative disorder such as Hodgkin’s Lymphoma has remained stable or even increased in HIV-positive adults since the introduction of ART and is ~11-fold higher than in the HIV-negative population (17). In this context, perinatally HIV infected children deserve particular attention, given their life-long exposure to chronic immune activation. It remains unknown whether early ART initiation during acute HIV infection followed by long-term virological suppression could control the levels of chronic immune activation and prevent abnormalities in the B-cell compartment. Longitudinally well characterized, adolescents living with perinatally acquired HIV-1 (PHIV) with no virologic or therapy failure and overall sustained virus suppression represent a unique opportunity to investigate this scientific question. Indeed, cohorts of children who started ART in infancy and maintain virological suppression are rare due to the rate of poor adherence observed in children. In the present work, we studied a European PHIV who have been treated with ART for >13 years with a documented history of viral suppression. We performed extensive characterization of the B-cell including phenotypic sign of B-cell hyperactivation (DN and ABCs) (18); T cell phenotyping with the added value of high-resolution proteomic analysis using mass spectrometry. Serum levels of anti-measles antibodies (Abs) were also analyzed as correlates of functional humoral immune response.

## Materials and Methods

### Study Population

The CARMA (Child and Adolescent Reservoir Measurements on early suppressive ART) cohort is part of the existing EPIICAL consortium (Early treated Perinatally HIV Infected individuals: Improving Children’s Actual Life) (19, 20), a multi-center, multi-cohort global collaboration primarily supported by PENTA foundation (Pediatric European Network for the Treatment of AIDS). CARMA included 40 perinatally HIV infected children (PHIV) with the following inclusion criteria: (1) start of ART within the 2^nd^ year of life; (2) ≥5 years of age; (3) viral suppression (<400 copies/mL) achieved in the first 12 months after initiation of ART and maintained for at least 5 years with 4 plasma viral load tests performed each year prior to enrolment; (4) a single viral load between 400 and 1000 c/mL (Spike) was permitted annually returning to less than 50 c/ml on next testing (within 3 months); (5) plasma viral load of <50 HIV-1 RNA copies/ml at enrolment. Wider characteristics of participants were described elsewhere (19) and relevant info is provided in **Table 1**. CD4 counts were collected at the hospital visits, and vaccination history was available from patients’ files.

**Table 1:** Characteristics of the study population.

	CARMA COHORT
N	40
Gender, M (%)	13/40 (32.5%)
Spike yes or no, n (%)	5/40 (13%)
At ART start	
Age median months (IQR)	4.1 (0.3-6.2)
CD4^+^ T cells median percentage (IQR)	30.5 (19.2-42.5)
Plasma HIV-1 RNA median copies/µL (IQR)	5.3 (4.1-5.7)
Time to suppression median months (IQR)	4.69 (2.52–6.26)
At analysis	
Age median years (IQR)	13.5 (8.7-16.6)
Time on ART median years (IQR)	13.5 (8.1-16.5)
CD4^+^ T cells median percentage (IQR)	41.0 (33.8-46.2)
caHIV-1 DNA median (copies/10^6^ PBMCs) (IQR)	48.4 (6.7-112.5)
caHIV-1 RNA (Pol) median (copies/10^6^ PBMCs) (IQR)	0.0 (0.0-1.4)
caHIV-1 RNA (LTR) median (copies/10^6^ PBMCs) (IQR)	2.7 (0.0-44.1)
anti-Measles IgG, median IU/l (IQR)	617 (411-936)
anti-Measles IgG, median years from vaccination (IQR)	5 (2-8)

M, male; IQR, interquartile range.

### Samples Collection

Plasma samples were obtained by centrifugation of EDTA-blood at 2000xg for 10’ and stored at -80°C until use. Peripheral blood mononuclear cells (PBMCs) were isolated using Ficoll density gradient centrifugation, resuspended in fetal bovine serum (FBS) supplemented with 10% dimethyl sulfoxide (DMSO), and stored in liquid nitrogen until use.

### B and T-Cell Phenotypic Analysis

**Figure 1:**
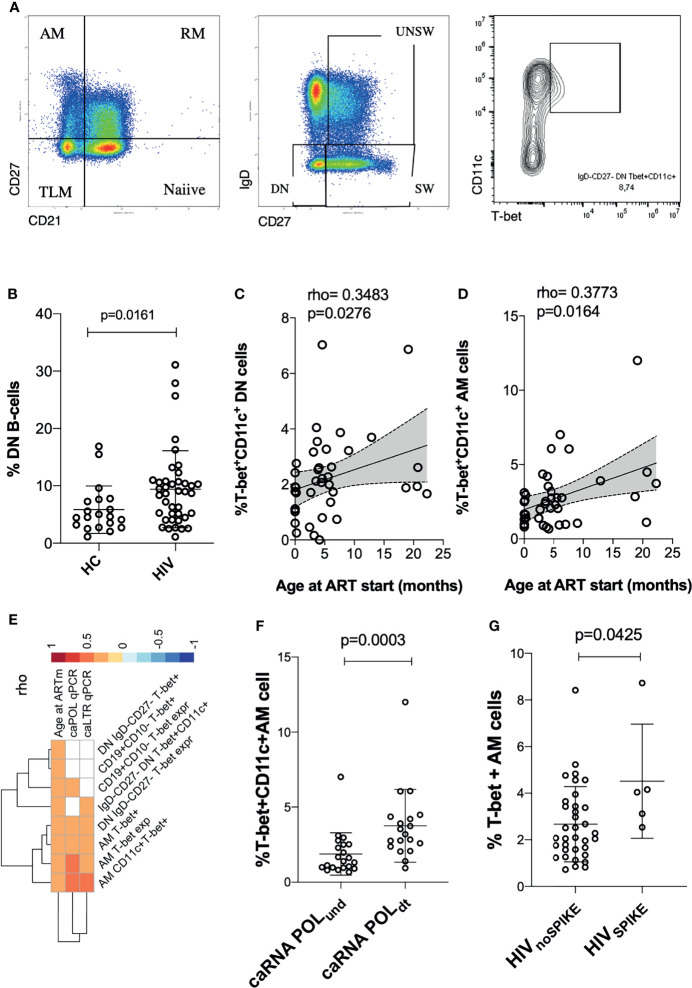
Time of ART initiation and cell associated HIV-1 RNA (caRNA) are associated with phenotypic signs of B cell hyperactivation. Gating strategy is shown in **(A)**; in **(B)** showed levels of DN in adolescents with perinatally aquired HIV-1 (HIV) and healthy controls (HC); **(C, D)** correlations between ABCs cells and age at ART start are shown; **(E)** correlation plot between viral correlates of recent replication and ABCs / exhausted T-cells are shown; differential analysis between levels of ABCs cells and caRNA or SPIKE being detected vs non-detected is shown in **(F, G)**. p values are calculated using Mann Whitney test in **(B, F, G)**. Spearman p values are shown in **(B–D)**. Significance was set at p>0.05. DN, double negative; AM, activated memory; SW, Switched B-cells (memory B-cells); UNSW, Unswitched B-cells; MFI, mean fluorescent intensity.

PBMCs from 40 PHIV were thawed, washed, and stained with the LIVE/DEAD fixable BV510 dead cell stain kit according to manufacturer’s protocol (Life Technologies, Carlsbad, CA), used to assess viability: positive cells were thus excluded from the analysis as they were considered as dead. For B-cell phenotype, after washing with PBS 10% FBS, cells underwent surface staining with the following monoclonal antibodies (mAbs, from BD Biosciences): CD3, CD10, CD16 (BV510), CD19 (APC-R700), CD21 (APC), CD27 (FITC), IgD (BV421), IgM (PE-CF594), IgG (BV605), CD11C (PC-7). Finally, stained cells were resuspended in 1% paraformaldehyde (PFA) and acquired using Stained cells were acquired on Cytoflex (Beckman Coulter, Brea, CA) and analysed with FlowJo v10.0.8 (Tree Star) software. Following surface staining fixing and permeabilization of cells (BD permeabilization solution II 1x), cells were stained with an anti T-bet BV650 (04-46, BD). For T-cell phenotype, LIVE/DEAD Fixable Blue Dead Cell Stain Kit from Thermo Fisher Scientific (Boston, MA) was used to detect and exclude dead cells. After washing with PBS 10% FBS, cells underwent surface staining with the following monoclonal antibodies as previously described (21): LAG3 BV650, TIGIT PE-Cy7, CD19 Alexa Fluor 700, HLA-DR PE, CCR7 FITC, CD38 BV711, PD-L1 BV711, PD-1 BV421, and CD8 PerCP from BioLegend (San Diego, CA); CD3 BUV496, CD4 APC-Cy7, CD4 APC-H7, PD-1 BV650, CXCR5 Alexa Fluor 647, and CD27 BV480 from BD Biosciences (San Jose, CA); and CD45RO PE-Cy5.5 from Beckman Coulter (Fullerton, CA). Finally, stained cells were resuspended in 1% paraformaldehyde (PFA) and acquired using Stained cells were acquired on a BD LSRFortessa (BD Biosciences) and analysis performed using FlowJo v10.0.8 (Tree Star) software. Gating strategies for B-cell phenotypes, T-bet and CD11c are provided in **Figure 1**. Gating strategies for T-cell analysis were shown previously (22). Positive cell gating was set using fluorescence minus one control. All the reagents were tested and titrated for optimum concentration before usage.

### Quantitative Total HIV-1 DNA Assay

Cell associated total HIV-1 DNA (caHIV-1 DNA)was quantified in PBMCs of 40 PHIV by real-time quantitative reverse transcription PCR (qRT-PCR) as previously described (23). All measurements were done in triplicates. Results are reported as copies of HIV-1 per million cells.

### Quantitative caHIV-1 RNA Assay

Cell associated HIV-1 RNA (caHIV-1 RNA) was quantified as described in (22). Briefly, Qiasymphony automated platform was used to isolate total cellular RNA (DSP virus/pathogen mini kit (Qiagen). RNA was further processed in an in-house assay using primers of previously validated assays (24, 25) to selectively amplify total (LTR) and unspliced (pol) ca-HIV-1 RNA *via* qRT-PCR. In order to express caHIV-1 RNA copies per 10^6^ PBMC, the caHIV-1 RNA measurements were normalized against cellular genes TBP1 and IPO8 expression.

### Plasma Proteomics Preparation and Analysis

Plasma proteomics data was produced using a High-performance liquid chromatography mass spectrometry (HPLC/MS) method as previously described (26). The sample processing employed an MStern blotting protocol previously developed and validated *in house* (27–30). In brief, 1 µL of plasma (~50 µg of proteins) was mixed in 100 µL of urea buffer. Following reduction and alkylation of the cysteine side chains, an amount of 15 µg of proteins was loaded on to a 96-well plate with a polyvinylidene fluoride (PVDF) membrane at the bottom (Millipore-Sigma), which had been previously activated and primed. Trypsinization of the proteins adsorbed to the membrane was achieved by incubation with the protease for 2h at 37°C. Resulting tryptic peptides were eluted off the membrane with 40% acetonitrile (ACN)/0.1% formic acid (FA). The peptides were subsequently cleaned-up using a 96-well MACROSPIN C18 plate (TARGA, The NestGroup Inc.). The samples were analysed on the same LC/MS system as the data-dependent acquisition (DDA) runs using identical LC parameters (45 minutes gradient, 59 minutes total runtime). The m/z range 375−1200, covering 95% of the identified peptide, was divided into 15 variable windows based on density, and the following parameters were used for the subsequent DIA analysis: resolution 35000 @ m/z 200, AGC target 3e6, maximum IT 120 ms, fixed first mass m/z 200, NCE 27. The DIA scans preceded an MS1 Full scan with identical parameters yielding a total cycle time of 2.4s. We use a previously published in house generated spectral library (26). All DIA data were directly analysed in Spectronaut v12.0.20491.18 (Biognosys, Switzerland). Standard search settings were employed, which included enabling dynamic peak detection, automatic precision nonlinear iRT calibration, interference correction, and cross run normalization (total peak area). All results were filtered by a q-value of 0.01 (corresponding to an FDR of 1% on the precursor and protein levels). Otherwise default settings were used.

### Anti-Measles IgG

Plasma Anti-Measles IgG titers were measured using EuroImmunAnti-Morbillo ELISA (IgG) (LOT E180111AE), following manufactures instruction. Results are given as UI/L.

### Statistical Analyses

**Figure 2:**
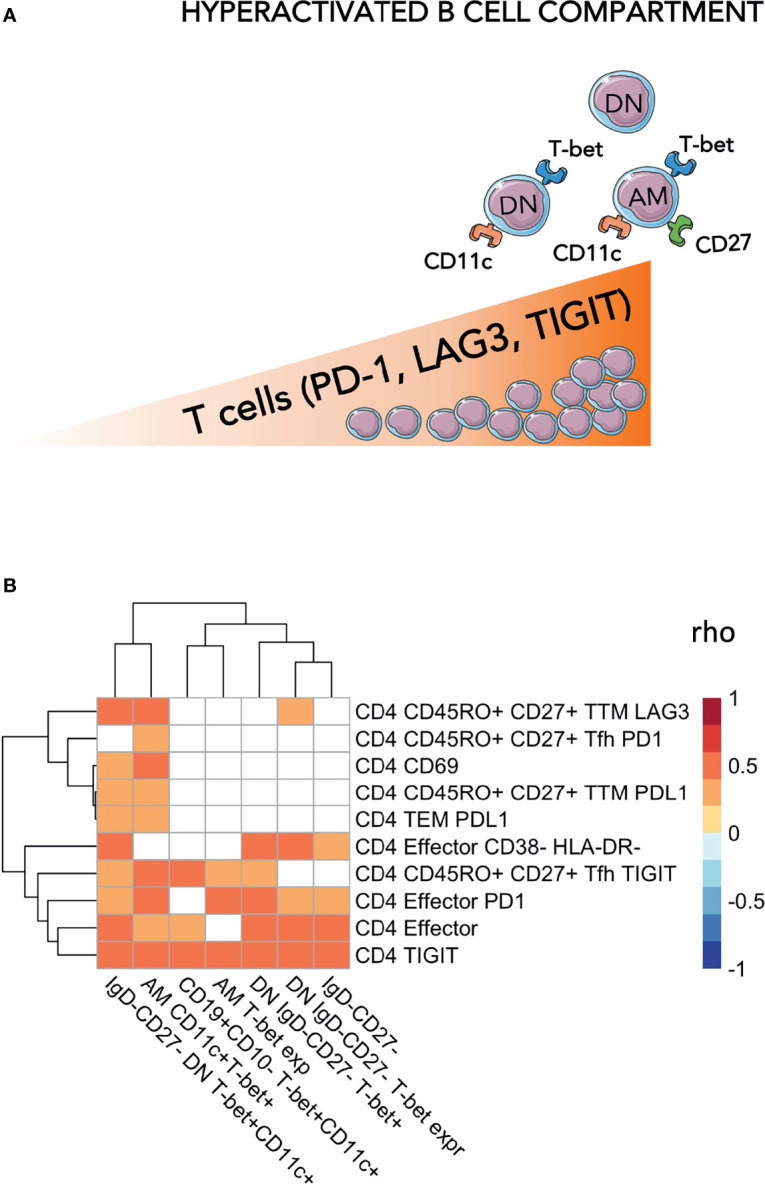
Levels of exhausted T-cells are positively associated with hyperactivated B-cells. In **(A)** a cartoon showing the main findings of the figures are pictured. In **(B)** Heatmap plot showing Spearman correlations between exhausted T-cells and hyperactivated B-cells. Only significant correlations are shown with red indicating positive correlations and Blue the negative ones. The colored scale going between 1 and -1 indicates the rho values. DN, double negative; AM, activated memory. Significance was set at p<0.05.

**Figure 3:**
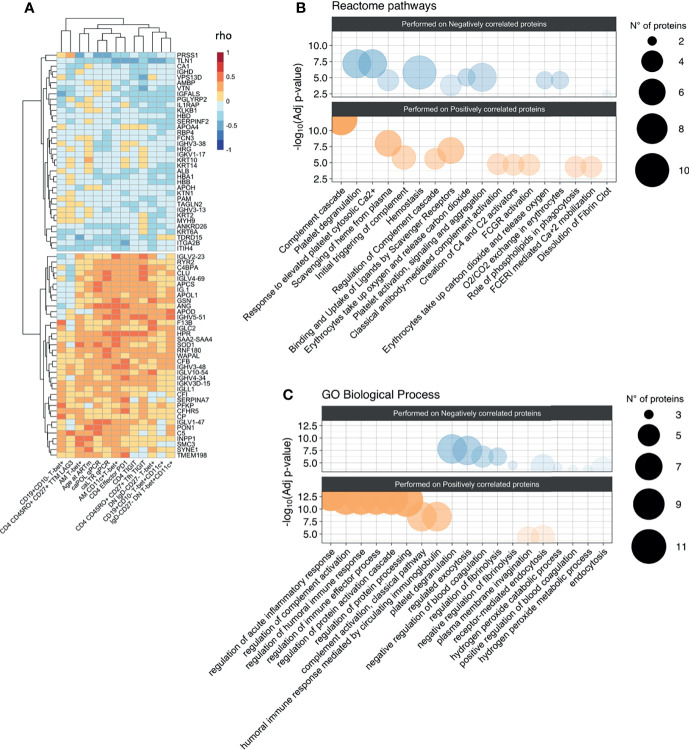
Association between proteomic profiling and levels of hyperactivated B-cells and exhausted T-cells. **(A)** Heatmap plot showing Spearman correlations between the 13 unfunctional features values and the abondance of the 73 plasma proteins belong to the two clusters identified in correlation matrix with all 338 proteins. Red indicates positive correlations and Blue negative ones. Bubble plots showing the top 10 Reactome pathways **(B)** and GO Biological Process **(C)** significantly enriched (Adjusted p-value < 0.05) in proteins positively (Pos) and negatively (Neg) correlated with the 13 unfunctional features. The proteins were separated into positively and negatively correlated based on the two clusters showed in the correlation heatmap in panel **(A)** Colors are related at the log_10_ adjusted p-value values and the circle diameter are related at the number of proteins for each term. Significance was set at p<0.05.

Between-group comparisons were performed using non-parametric U-Mann-Whitney test for continuous variables or Fisher’s exact test for categorical variables. Spearman correlation (rho) was used to describe the association between continuous variables. Proteins and cell populations with >70% zero values or >50% missing data were omitted from heatmaps. To focus on single associations (**Figures 1D**, **2B**, **3A**), only statistically significant correlations (p-values <0.05) were shown. In other cases, to highlight clustering patterns all correlations were shown (**Figure 3A** and **Supplementary Figure 1**). The chromatic scale is proportional to the Spearman correlation, using red for positive correlations (rho > 0) and blue for negative ones (rho < 0). To investigate the biological role of the proteins belonging to the two clusters (**Figure 3A**), a pathway enrichment analysis in Reactome 2016 and GO Biological Process 2021 databases was performed using the R package “enrichr” v3.0 (31). Statistical analyses were performed using R (version 4.1.1) or GraphPad Prism 6.0 software (San Diego, CA).

## Results

### Study Cohort

Patient characteristics are shown in **Table 1**. Overall, we analyzed 40 PHIV (males 13/40, 32.5%) that started ART at a median of 4.1 months (IQR 0.3-6.2), achieved virological suppression after a median of 4.69 (2.52–6.26), and were successfully on ART for median 13.5 years (8.1-16.5). We measured caHIV-1 DNA (caHIV-1 DNA median 48.8 copies/10^6^ PBMC), caHIV-1 RNA in the Pol and LTR regions. Overall, 5/40 (13%) had experienced up to two transient elevations of the plasma HIV-1 RNA load (herein referred to as ‘Spike’, HIV-1 VL between 400-999 c/mL, returning to VL <50 c/ml at next blood draw) (**Table 1** and **Figure 1**).

### Time of ART-Start and caRNA Are Associated With Phenotypic Signs of B Cell Hyperactivation

We performed an extensive immune phenotyping focusing on the B-cell compartment (gating strategy shown in **Figure 1A**). We found that DN B-cells were expanded in HIV-infected as compared to age-gender matched (N=20; 65% female; median age= 11.3 years, IQR=7.6-16.1) HIV-unifected individuals (historical internal data from the clinical immunology lab, **Figure 1B**) (7, 32, 33). We further explored the expression of T-bet+CD11c+ as sign of B-cell hyperactivation. The proportion of DN and AM expressing both T-bet and CD11c were positively associated with the time of ART initiation, with expansion of T-bet^+^CD11c^+^ DN B cells (p=0.03, **Figure 1C**) and T-bet^+^CD11c^+^ AM B cells (p=0.02, **Figure 1D**) in those individuals with delayed ART initiation. We further explored if these ABCs were associated with the HIV-reservoir. Whereas caHIV-1 DNA showed no association, both total caHIV-1 RNA (LTR) and unspliced caHIV-1 RNA (Pol) demonstrated a positive association with the ABCs (**Figure 1E**). caHIV-1 RNA was associated with B cells, AM and DN expressing T-bet^+^ alone or together with CD11c, with higher levels of these B-cell populations present in individuals with detectable ongoing virus expression (**Figures 1E, F**). We further stratified the study participants by those who did (group I= 5) or did not (group II= 35) experience Spikes (**Figure 1G**). Group I had significantly higher levels of AM T-bet+ cells compared to group II (p=0.04, **Figure 1G**). These data showed that age at ART initiation is strongly correlated with levels of B-cell hyperactivation in PHIV as well as ongoing HIV-1 replication.

### Individuals With Expansion of Phenotypic Signs of B Cell Hyperactivation Have Elevated Levels of Exhausted T-Cells

We then explored whether the levels of DN and ABCs were associated with levels of exhausted T-cells. Within the ABCs, we included T-bet^+^CD11c^+^, T-bet+ only B-cells, or levels of T-bet (MFI) within the whole B compartment as well as within the ‘namely exhausted’ phenotypes (AM and DN). In assessing the T-cell compartment, we focused on populations expressing exhaustion biomarkers (showed in **Figure 2A**). Overall, correlation analysis demonstrated direct positive associations between B and T cells, suggesting that a certain extent of immune hyperactivation/exhaustion persisted in different cellular populations, even many years after successful treatment and virological control (**Figure 2B** and **Supplementary Table 1**). In particular: %CD4 TIGIT+ were associated with %DN (p=0.008), %DN T-bet+CD11c+ (p=0.0002) and %AM T-bet+CD11c+ (p=0.002) and %CD4 PD-1 were associated with %DN (p=0.048), %DN T-bet+CD11c+ (p=0.039) and %AM T-bet+CD11c+ (p=0.006). Furthermore, AM T-bet^+^CD11c^+^ was associated with PD-1 expression on CD4 effector (p=0.006). Similarly, LAG3 expression on transitional memory (TTM) demonstrated a strong association with AM (p=0.002) and DN (p=0.003) expressing both T-bet and CD11c. These data demonstrated that hyperactivation and exhaustion persists simultaneously in both B and T cell compartments, even after >10 years of ART.

### Proteomic Profiles Associated With B Cell Hyperactivation

To assess whether humoral/soluble factors might correspond to hyperactivated phenotypes, we performed liquid chromatography/mass spectrometry-based proteomics, detecting 338 plasma proteins (26). The distinct immunological, virological, and clinical features were correlated to the whole plasma proteomic profile (**Supplementary Figure 1**). Two distinct clusters were initially identified, which were negatively (36 proteins) or positively (37 proteins) associated with phenotypic signs of B cell hyperactivation and exhausted T-cells (**Figure 3A**). Such protein clusters were further interrogated for their biological role by enrichment analysis on Reactome and Gene Ontology (GO) biological processes databases (**Figure 3B**). Most of the ‘positively associated’ factors were classified within the immune inflammation and complement cascade (bottom panels, **Figures 3B, C**). Indeed, amyloid P component in serum (APCS) and clustering (CLU), both involved in apoptotic, aging and tumor progression processes (GO:0002673) together with complement cascade molecules such as C5, CFI, C4BPA, CFB (R-HSA-173623) were positively associated to selected features of phenotypic signs of B cell hyperactivation and exhausted T-cells (**Supplementary Table 2**). In addition, also proteins of light and heavy chain of immunoglobulins, involved in humoral immune response pathway (GO:0002920) such as IGLV1-47, IGHV4-34, IGLV2-23, IGHV3-48 showed a positive association. Enrichment analysis performed on negatively correlated proteins, showed no association with inflammatory pathways but only with processes involved in coagulation. Indeed, proteins such as APOH, SERPINF2, HRG, involved in pathways of negative regulation of blood coagulation (GO:0030195) and platelet degranulation (R-HSA-76002), were negatively associated with features of phenotypic signs of B cell hyperactivation or exhausted T-cells (**Supplementary Table 2**).

### Expansion of ABCs Is Associated With B-Cell Dysfunctionality in PHIV

**Figure 4:**
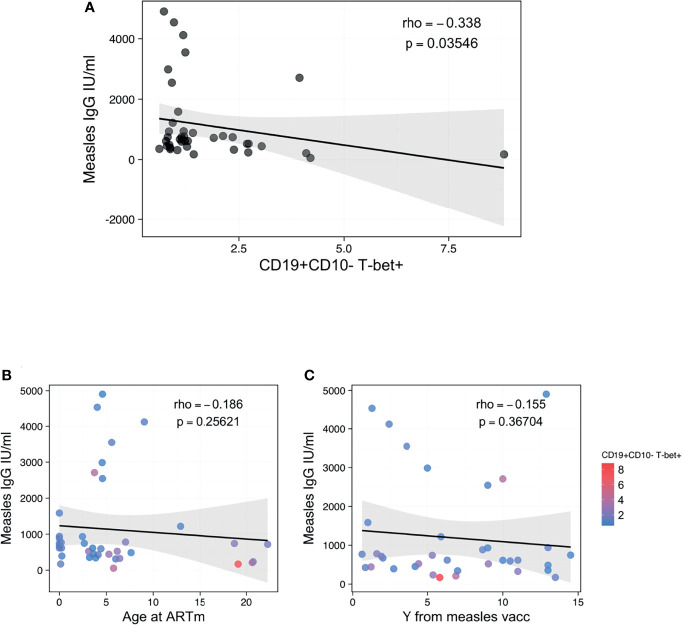
Association between ABCs and anti-measles humoral response. **(A)** Spearman correlation between CD19+CD10- B-cells T-bet+ and anti-Measle plasma IgG titers, with rho and p defining the statistical significance. **(B, C)** Spearman correlation between anti-Measle plasma IgG titers and Age at ART in m and years from measles vaccination, respectively, with rho and p defining the statistical significance. Color dots show the distribution of CD19+CD10- B-cells T-bet+. Significance was set at p<0.05.

We further assessed whether the presence of ABCs could affect the functionality of the B-cell compartment to maintain immunological memory against vaccinations, such as measles. Interestingly, the proportion of B-cells expressing T-bet only demonstrated a negative association with B-cells’ capacity to maintain immunological memory to measles vaccination (**Figure 4**). Higher levels of CD19+CD10-T-bet+ B cells were associated with reduced plasma concentrations of anti-measles specific IgG (**Figure 4A**, rho=-0.338, p=0.03546). Of note, this association was strong regardless of the time of ART initiation (**Figure 4B**) or timing from the last booster vaccination (**Figure 4D**).

## Discussion

In this work, we studied the determinants of B cell compartment hyperactivation in a cohort of PHIV after over 10 years of suppressive ART, started within 2 years of age. Higher levels of DN and ABCs were found in individuals who started ART later, with higher levels of HIV caRNA, levels of exhausted T-cells and with specific proteomic profiles. The expansion of ABCs appeared to have a direct impact on the ability of these patients to maintain vaccine induced immunity over time.

PHIV children, particularly younger ones, are immunologically distinct from adults with regards to plasticity and immune regulation, resulting in a lower immune activation state (34). Since chronic immune activation in treated HIV infection is probably driven by residual HIV replication (35, 36), an initiation of ART early in life followed by sustained suppression of the viral replication should minimize levels of chronic activation (37). In this work, we show that perinatally infected adolescents starting ART early in life present lower frequency of DN and ABCs compared to those starting ART later.

We next explored the virological determinants of the expansion of ABCs in PHIV, with the rational that low level virus replication may drive chronic immune inflammation and consequentially driving the expansion of distinct B-cells subset. Total HIV-1 DNA showed no correlation, probably reflecting the fact that the contribution of the replication-competent virus is diluted within the entire integrated virus reservoir, which is mainly inactive (38). Further, we explored the markers of recent virus replication. Both spliced and unspliced HIV-1 caRNA were strongly associated with levels of ABCs. Spliced HIV-1 RNA may reflect abortive HIV-replication, with only a minor part being released as virus protein or exosome-associated fragments of RNA that can still trigger immune activation (39). In contrast, the unspliced HIV-1 RNA is thought to predict the replicative-competence of the virus reservoirs and has been associated with virologic failure and markers of immune activation in elite controllers (37, 40, 41), besides been recently proposed as a predictive marker of viral rebound (42). In addition, the frequency of ABCs was higher in those PHIV who experienced HIV-1 spikes in absence of virologic failure. The association between expansion of atypical B cell subsets, caRNa and viral Spikes is consistent with the hypothesis HIV-1 replication and virus particle release fuels chronic immune activation (43).

Multiple mechanisms likely underpin the association between caHIV-1 RNA and B-cell compartment hyperactivation: 1) HIV-1 particles can interact directly with B cells surface-bound *via* the CD21 receptor with complement 3 (C3) fragment both in peripheral blood and lymph nodes of HIV-1 patients (44); and 2) B-cells may function as Antigen Presenting Cells (APC) taking direct contact with follicular T-cells to trigger an anti-HIV-response. Transient circulation of few HIV-1 virus, despite continuous virologic control, could sustain a state of chronic immune activation affecting both B and T cells compartment that could result in expansion of signatures associated with hyperactivation, exhaustion, and atypical phenotypes (22, 45). Consistent with this hypothesis, our results showed that ABCs existed simultaneously with T-cell expressing PD-1, TIM-3 and LAG-3 which are inhibitory receptors that are found to be increased on the T-cell surface as a consequence of persistent activation and described as markers of cells exhaustion (46). Furthermore, T-bet+CD11c+ B-cells were associated with exhausted Tfh in accordance with other models of chronic antigenic stimulation such as auto-immune diseases (47), such as lupus. In fact, the excessive T-bet+CD11c+ ABCs (48) not only contribute to the production of auto-Abs but they also promote aberrant Tfh cell differentiation resulting in inadequate affinity-based germinal center B-cell selection and Ab-affinity maturation in lupus mouse models.

We next address if signs of chronic immune activation could be identified within our cohort’s repertoire of plasma soluble factors, thus supporting the expansion of atypical B-cells (44). Our untargeted proteomic analysis showed that proteins involved in pro-inflammatory and complement activation processes were positively associated with ABCs. While it was previously shown that the initiation of ART during the acute phase of the infection in HIV-infected adults reduced levels of immune activation (35, 36), we here show that early initiation of ART in PHIV does not prevent the persistence of correlates of chronic immune activation, despite a history of long-term viral suppression (>10 years). Specifically, APCS and CLU, both involved in processes of cell apoptosis, inflammation, and lymphoproliferative processes (49–51) were positively associated with caHIV-RNA, immune checkpoint-inhibitors (TIGIT and PD1 on T cells) and ABCs. Accordingly, such proteins were shown to be higher in HIV-infected adults experiencing a poor immune reconstitution and disease progression despite viral control (52). Furthermore, the positive associations between activated or T-bet+/CD11c+ and of light and heavy chain of immunoglobulins (GO:0002920) (IGLV1-47, IGHV4-34, IGLV2-23, IGHV3-48) further showed a residual B-cell activation and dysfunction persists despite long term viral suppression as also recently showed in adults (53).

Proteomics further showed that the complement cascade activation pathway was enriched in proteins positively associated with immunological hyperactivation features including CLU. As previously demonstrated, the complement activation contributes to a chronic pro-inflammatory environment even in well-controlled HIV infected adults (54). Whereas the activation of the complement cascade during acute HIV infection is largely *via* activation of the classical pathway (52, 55), recent studies highlight how complement factors bind IgG3 on exhausted B cell subsets (TLM) in HIV-positive individuals (44, 56). In line with this evidence, our results showed a positive association of both caHIV-RNA and ABCs (T-bet+CD11c+ DN and AM) with plasma complement cascade proteins.

Correlation analysis further revealed an association of proteins involved in coagulation processes with features of hyperactivation. As previously shown in adults, a pro-coagulative imbalance, partially resolved by ART initiation during the acute infection (35, 36) and persisting over time in HIV infected adults (57), was confirmed in our cohort where a regulation of fibrinolysis was negatively associated with ABCs and exhausted T-cells. Overall, plasma proteomic profiling may suggest that the persistence of complement cascade perturbation, rather than inflammatory and coagulation proteins, may contribute to B -cell exhaustion and signs hyperactivation in long term virally controlled (>10 years) PHIV.

Finally, we tested if the of signs of B cell hyperactivation could reflect an impairment of the maintenance of the humoral response towards childhood vaccination, such as measles immunization which should be maintained throughout life in physiological conditions. Recent findings on lymph nodes suggest that chronic immune activation favors T-bet expression on B cells which is detrimental to the generation of effective humoral immunity, both as a result of accumulation outside GC and induction of immune-regulating mechanisms (58). In line with this, we found that a higher proportion of peripheral B-cells expressing T-bet were negatively associated with anti-measles serum IgG levels. These data are not biased by the natural Ab decay because patients were analyzed at similar median years from vaccination. While our study featured multiple strengths, as with all research it also had some limitations to consider. The small size of the CARMA cohort limited the power of correlation analysis to detect associations; it would be interesting to expand the immunological profiling to a larger cohort. The lack of a control group of exposed uninfected HIV individuals and potentially of another group that started therapy after 2 years of age, to deeply investigate the impact of late ART start. The data on the association between % T-bet+ B-cells and weaning of anti-Measles antibodies titers appear to have weak statistical power that warrant further confirmation in bigger cohorts. Cross-sectional study design.

## Conclusions

In conclusion, our study demonstrated that B cell hyperactivation is still visible despite >10 years of suppressive ART which can negatively affect the capacity of B-cell compartment to maintain a vaccine-induced functional Ab response. Further studies are needed in order to confirm whether hereby described signatures of hyperactivation/inflammation can inform simplified methods to stratify the risk of disease progression or of development lymphoproliferative disorders in cohorts of long-term suppressed PHIV.

## Data Availability Statement

The datasets presented in this study can be found in online repositories. The names of the repository/repositories and accession number(s) can be found below: ProteomeXchange, accession no: PXD031908.

## Ethics Statement

This is a multi-center study which includes the following institutions: Bambino Gesù Children’s Hospital (OPBG, Rome, Italy), University of Padua (Padova, Italy), University Hospital 12 de Octubre (Madrid, Spain), Hospital Gregorio Marañón (Madrid, Spain), Imperial College Healthcare NHS Trust (London, UK), Great Ormond Street Hospital (London, UK), Brighton and Sussex University Hospitals (Brighton, UK). Each recruiting site received approval by local ethic committees (19). Study participants or their legal guardians gave written informed consent in accordance with the Declaration of Helsinki.

## Epiical Consortium

Mark Cotton, Shaun Barnabas, Thanyawee Puthanakit, Louise Kuhn, Andrew Yates, Avy Violari, Kennedy Otwombe, Paula Vaz, Maria Grazia Lain, Tacilta Nampossa, Denise Naniche, Sheila Fernandez-Luis, Elisa Lopez, Holly Peay, Moira Spyer, Vincent Calvez, Anne-Genevieve Marcelin, Maria Angeles Munoz, Annalisa Dalzini, Raffaella Petrara, Kathleen Gartner, Lesley De Armas, Pahwa Rajendra, Suresh Pallikkuth, Deborah Persaud, Nicolas Chomont, Mathias Lichterfeld, Silvia Faggion, Daniel Gomez Pena, Andrea Oletto, Alessandra Nardone, Paola Zangari, Silvia Di Cesare, Chiara Medri, Olga Kolesova, Carla Paganin, William James, Inger Lindfors - Rossi, Shrabon Samiur Hassan, Francesca Mazzetto, Hellen Akisinku, Musakanya Chingandu, Francesca Rocchi, Ilaria Pepponi, Rob J. De Boer, Juliane Schroter, Viviana Giannuzzi, Andrew Yates, Sinead Morris.

## Author Contributions

PP, AR, GRP, NC conceived the paper design, analysed, and discussed data and wrote the manuscript; PP, NC, CF, MDP, AT, PRoj, AB, CG, PR designed the study, enrolled patients, collected and managed clinical data; AR, GRP, SD-R, performed statistical analysis; AR, GRP, NC, SR, KS, AO, ADR, EN, EM, BF performed experiments; NK, HS, KL, PG, PRos, OL, SP, PP supervised the work. All authors read, critically revised and approved the manuscript.

## Funding

This study was supported by PENTA-ID Foundation (http://penta-id.org/), funded through an independent grant by ViiV Healthcare UK. PP and SP were supported by the NIH grant R01AI127347-05. Work performed at the Laboratory Sciences Core of the Miami was supported by CFAR (P30AI073961) and by the following NIH Co-Funding and Participating Institutes and Centres: NIAID, NCI, NICHD, NHLBI, NIDA, NIMH, NIA, NIDDK, NIGMS, FIC, and OAR. The funders had no role in study design, data collection and analysis, decision to publish, or preparation of the manuscript.

## Conflict of Interest

The authors declare that the research was conducted in the absence of any commercial or financial relationships that could be construed as a potential conflict of interest.

## Publisher’s Note

All claims expressed in this article are solely those of the authors and do not necessarily represent those of their affiliated organizations, or those of the publisher, the editors and the reviewers. Any product that may be evaluated in this article, or claim that may be made by its manufacturer, is not guaranteed or endorsed by the publisher.

## Supplementary Material

Supplementary Figure 1 Heatmaps showing Spearman correlations between abundance of 338 plasma proteins and 13 selected unfunctional features. Red indicates positive correlations and Blue negative ones.

Supplementary Table 1 Overview of p and rho values describing association between hyperactivated B-cells and exhausted T-cells shown in **Figure 2**.

Supplementary Table 2 Overview of proteins within the Reactome and GO biological process pathways associated with hyperactivated B-cells and exhausted T-cells.
