# Supplementary material for: Determinants of B-Cell Compartment Hyperactivation in European Adolescents Living With Perinatally Acquired HIV-1 After Over 10 Years of Suppressive Therapy
Source: Front Immunol. 2022 Mar 31;13:860418. doi: 10.3389/fimmu.2022.860418 (PMC9009387; doi:10.3389/fimmu.2022.860418)
Supplement: Supplementary Figure 1 — Heatmaps showing Spearman correlations between abundance of 338 plasma proteins and 13 selected unfunctional features. Red indicates positive correlations and Blue negative ones. [file DataSheet_1.pdf]

Supplementary Fig.1

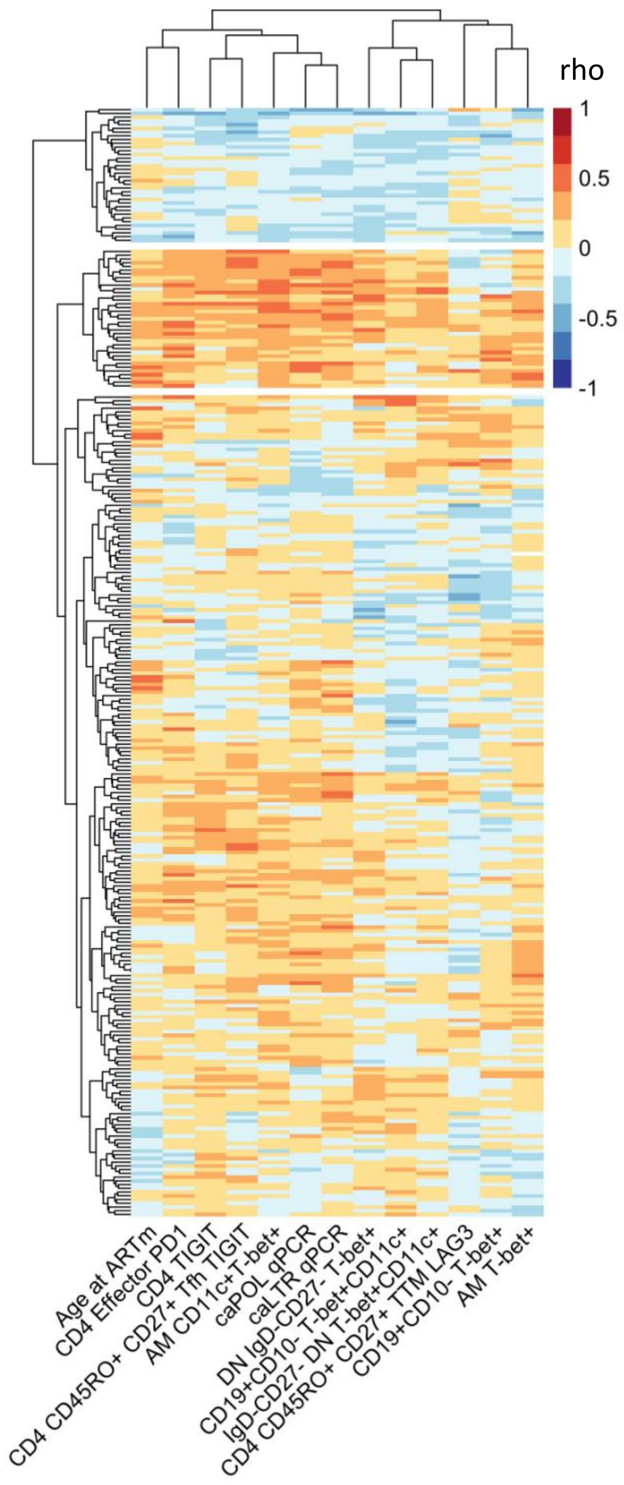

Supplementary Table 1

**P values**	CD19+CD10- T-bet+CD11c+	IgD-CD27- DN T-bet+CD11c+	AM CD11c+T-bet+	DN IgD-CD27- T-bet+	DN IgD-CD27- T-bet expr	AM T-bet exp	IgD-CD27-
CD4 CD45RO+ CD27+ TTM LAG3	0.461785565	0.004998415	0.0109684	0.277308488	0.021389135	0.192170669	0.186771485
CD4 CD69	0.919705883	0.046704979	0.01265367	0.50690658	0.187838184	0.211046551	0.94324879
CD4 CD45RO+ CD27+ TTM PDL1	0.743632288	0.032254947	0.022989327	0.466388794	0.08269117	0.169195972	0.098805957
CD4 TEM PDL1	0.223082784	0.04300951	0.020614009	0.156102816	0.223748423	0.407866115	0.099966572
CD4 CD45RO+ CD27+ Tfh PD1	0.064706569	0.302856523	0.049687336	0.477797321	0.142906932	0.233034157	0.15279861
CD4 Effector CD38- HLA-DR-	0.062731004	0.00775711	0.090184541	0.001422804	0.011897081	0.079223142	0.033816508
CD4 Effector	0.023755404	0.002439781	0.0395915	0.002754557	0.007045795	0.059753248	0.009292235
CD4 TIGIT	0.002732047	0.000279364	0.002070491	0.006341559	0.006323544	0.007768473	0.008985975
CD4 CD45RO+ CD27+ Tfh TIGIT	0.007941634	0.023878661	0.015329827	0.016272815	0.058745236	0.031669457	0.122769808
CD4 Effector PD1	0.065957926	0.039291289	0.006647288	0.000281472	0.040581401	0.012364855	0.048780452

**rho**	CD19+CD10- T-bet+CD11c+	IgD-CD27- DN T-bet+CD11c+	AM CD11c+T-bet+	DN IgD-CD27- T-bet+	DN IgD-CD27- T-bet expr	AM T-bet exp	IgD-CD27-
CD4 CD45RO+ CD27+ TTM LAG3	NS	0.457746034	0.41902678	NS	0.382301836	NS	NS
CD4 CD69	NS	0.343494538	0.423135697	NS	NS	NS	NS
CD4 CD45RO+ CD27+ TTM PDL1	NS	0.357597999	0.378089601	NS	NS	NS	NS

CD4 TEM PDL1	NS	0.339168493	0.384437151	NS	NS	NS	NS
CD4 CD45RO+ CD27+ Tfh PD1	NS	NS	0.329535947	NS	NS	NS	NS
CD4 Effector CD38- HLA-DR-	NS	0.436635137	NS	0.511716235	0.414761426	NS	0.354637324
CD4 Effector	0.376158602	0.489606797	0.344574591	0.484423279	0.441367838	NS	0.427569343
CD4 TIGIT	0.484776313	0.570601112	0.496492245	0.446475701	0.44661263	0.436562601	0.42927018
CD4 CD45RO+ CD27+ Tfh TIGIT	0.435468298	0.375852751	0.401107036	0.397811394	NS	0.358738334	NS
CD4 Effector PD1	NS	0.345067251	0.444201313	0.570352734	0.342971273	0.41271885	0.330780617

Supplementary Table 2

Reactome pathways - Performed on Negatively correlated proteins

Term	Adjusted.P.value	Proteins
Platelet degranulation Homo sapiens R-HSA-114608	6.88744E-08	ITIH4, APOH, ITGA2B, SERPINF2, ALB, HRG, TLN1
Response to elevated platelet cytosolic Ca2+ Homo sapiens R-HSA-76005	6.88744E-08	ITIH4, APOH, ITGA2B, SERPINF2, ALB, HRG, TLN1
Hemostasis Homo sapiens R-HSA-109582	1.57513E-06	ITIH4, APOH, ITGA2B, SERPINF2, ALB, HBB, HBD, HRG, TLN1, KLKB1
Platelet activation, signaling and aggregation Homo sapiens R-HSA-76002	8.94125E-06	ITIH4, APOH, ITGA2B, SERPINF2, ALB, HRG, TLN1
Erythrocytes take up oxygen and release carbon dioxide Homo sapiens R-HSA-1247673	8.94125E-06	CA1, HBB, HBA1
Erythrocytes take up carbon dioxide and release oxygen Homo sapiens R-HSA-1237044	2.49667E-05	CA1, HBB, HBA1
O2/CO2 exchange in erythrocytes Homo sapiens R-HSA-1480926	2.49667E-05	CA1, HBB, HBA1
Scavenging of heme from plasma Homo sapiens R-HSA-2168880	2.56245E-05	AMBP, ALB, HBB, HBA1
Binding and Uptake of Ligands by Scavenger Receptors Homo sapiens R-HSA-2173782	0.000154953	AMBP, ALB, HBB, HBA1
Dissolution of Fibrin Clot Homo sapiens R-HSA-75205	0.003639893	SERPINF2, HRG

Reactome pathways - Performed on Positively correlated proteins

Term	Adjusted.P.value	Proteins
Complement cascade Homo sapiens R-HSA-166658	1.6472E-12	IGLV4-69, C5, CFI, IGLV2-23, C4BPA, IGLC2, CLU, CFB, IGLV10-54
Scavenging of heme from plasma Homo sapiens R-HSA-2168880	1.02257E-08	IGLV4-69, IGLV2-23, HPR, IGLC2, APOL1, IGLV10-54
Binding and Uptake of Ligands by Scavenger Receptors Homo sapiens R-HSA-2173782	1.29901E-07	IGLV4-69, IGLV2-23, HPR, IGLC2, APOL1, IGLV10-54
Initial triggering of complement Homo sapiens R-HSA-166663	1.53583E-06	IGLV4-69, IGLV2-23, IGLC2, CFB, IGLV10-54
Regulation of Complement cascade Homo sapiens R-HSA-977606	2.53246E-06	C5, CFI, C4BPA, CFB
Classical antibody-mediated complement activation Homo sapiens R-HSA-173623	1.70347E-05	IGLV4-69, IGLV2-23, IGLC2, IGLV10-54
FCGR activation Homo sapiens R-HSA-2029481	2.17615E-05	IGLV4-69, IGLV2-23, IGLC2, IGLV10-54
Creation of C4 and C2 activators Homo sapiens R-HSA-166786	2.17615E-05	IGLV4-69, IGLV2-23, IGLC2, IGLV10-54
Role of phospholipids in phagocytosis Homo sapiens R-HSA-2029485	4.38779E-05	IGLV4-69, IGLV2-23, IGLC2, IGLV10-54
FCERI mediated Ca+2 mobilization Homo sapiens R-HSA-2871809	4.50569E-05	IGLV4-69, IGLV2-23, IGLC2, IGLV10-54

GO Biological Process - Performed on Negatively correlated proteins

Term	Adjusted.P.value	Proteins
platelet degranulation (GO:0002576)	1.58619E-08	ITIH4, APOH, ITGA2B, SERPINF2, ALB, TAGLN2, HRG, TLN1
regulated exocytosis (GO:0045055)	3.29263E-08	ITIH4, APOH, ITGA2B, SERPINF2, ALB, TAGLN2, HRG, TLN1
negative regulation of blood coagulation (GO:0030195)	5.44959E-07	VTN, APOH, SERPINF2, HRG, KLKB1
regulation of fibrinolysis (GO:0051917)	5.44959E-07	APOH, SERPINF2, HRG, KLKB1
negative regulation of fibrinolysis (GO:0051918)	3.11665E-05	APOH, SERPINF2, HRG
receptor-mediated endocytosis (GO:0006898)	5.70694E-05	AMBP, IGKV1-17, ALB, IGHV3-13, HBB, HBA1
hydrogen peroxide catabolic process (GO:0042744)	9.58743E-05	HBB, APOA4, HBA1
positive regulation of blood coagulation (GO:0030194)	0.000221947	APOH, SERPINF2, HRG
hydrogen peroxide metabolic process (GO:0042743)	0.000231815	HBB, APOA4, HBA1
endocytosis (GO:0006897)	0.000238417	AMBP, IGKV1-17, ALB, IGHV3-13, HBB, HBA1

GO Biological Process - Performed on Positively correlated proteins

Term	Adjusted.P.value	Proteins
regulation of acute inflammatory response (GO:0002673)	8.01976E-14	APCS, C5, IGLV1-47, CFI, IGHV4-34, IGLV2-23, CFHR5, C4BPA, IGHV3-48, CLU, CFB
regulation of protein activation cascade (GO:2000257)	6.35311E-13	C5, IGLV1-47, CFI, IGHV4-34, IGLV2-23, CFHR5, C4BPA, IGHV3-48, CLU, CFB
regulation of complement activation (GO:0030449)	6.35311E-13	C5, IGLV1-47, CFI, IGHV4-34, IGLV2-23, CFHR5, C4BPA, IGHV3-48, CLU, CFB
regulation of humoral immune response (GO:0002920)	6.35311E-13	C5, IGLV1-47, CFI, IGHV4-34, IGLV2-23, CFHR5, C4BPA, IGHV3-48, CLU, CFB
regulation of immune effector process (GO:0002697)	6.35311E-13	C5, IGLV1-47, CFI, IGHV4-34, IGLV2-23, CFHR5, C4BPA, IGHV3-48, CLU, CFB
regulation of protein processing (GO:0070613)	1.73448E-12	C5, IGLV1-47, CFI, IGHV4-34, IGLV2-23, CFHR5, C4BPA, IGHV3-48, CLU, CFB
complement activation, classical pathway (GO:0006958)	3.30081E-09	APCS, IGHV5-51, IGLV1-47, IGLL1, IGHV4-34, IGLV2-23, IGHV3-48, IGLC2
humoral immune response mediated by circulating immunoglobulin (GO:0002455)	3.30081E-09	APCS, IGHV5-51, IGLV1-47, IGLL1, IGHV4-34, IGLV2-23, IGHV3-48, IGLC2
receptor-mediated endocytosis (GO:0006898)	5.13899E-05	IGLV1-47, IGHV4-34, IGLV2-23, IGHV3-48, HPR, APOL1
plasma membrane invagination (GO:0099024)	5.13899E-05	IGHV5-51, GSN, IGLL1, IGHV3-48, IGLC2
